# The WRKY Transcription Factor Genes in Eggplant (*Solanum melongena* L.) and Turkey Berry (*Solanum torvum* Sw.)

**DOI:** 10.3390/ijms16047608

**Published:** 2015-04-07

**Authors:** Xu Yang, Cao Deng, Yu Zhang, Yufu Cheng, Qiuyue Huo, Linbao Xue

**Affiliations:** 1 College of Horticulture and Plant Protection, Yangzhou University, Yangzhou 225009, China; E-Mails: yangxu@yzu.edu.cn (X.Y.); 15161883742@163.com (Y.Z.); 15506101223@163.com (Q.H.); lbxue@yzu.edu.cn (L.X.); 2 DNA Stories Bioinformatics Services Co., Ltd., Chengdu 610000, China; E-Mail: brentcaodeng@gmail.com

**Keywords:** *Solanum torvum* sw., *Solanum melongena* L., WRKY transcriptional factor

## Abstract

WRKY transcription factors, which play critical roles in stress responses, have not been characterized in eggplant or its wild relative, turkey berry. The recent availability of RNA-sequencing data provides the opportunity to examine WRKY genes from a global perspective. We identified 50 and 62 WRKY genes in eggplant (SmelWRKYs) and turkey berry (StorWRKYs), respectively, all of which could be classified into three groups (I–III) based on the WRKY protein structure. The SmelWRKYs and StorWRKYs contain ~76% and ~95% of the number of WRKYs found in other sequenced asterid species, respectively. Positive selection analysis revealed that different selection constraints could have affected the evolution of these groups. Positively-selected sites were found in Groups IIc and III. Branch-specific selection pressure analysis indicated that most WRKY domains from SmelWRKYs and StorWRKYs are conserved and have evolved at low rates since their divergence. Comparison to homologous WRKY genes in Arabidopsis revealed several potential pathogen resistance-related SmelWRKYs and StorWRKYs, providing possible candidate genetic resources for improving stress tolerance in eggplant and probably other Solanaceae plants. To our knowledge, this is the first report of a genome-wide analyses of the SmelWRKYs and StorWRKYs.

## 1. Introduction

Eggplant (*Solanum melongena* L.) is the third most agriculturally important crop from the genus *Solanum* after potato (*S. tuberosum*) [1] and tomato (*S. lycopersicum*) [2]. In 2011, 46.8 million tons of eggplant were produced in the top four producing countries, namely China (27.7 million tons), India (11.8 million tons), Egypt (1.1 million tons) and Turkey (8.2 million tons), according to the Food and Agriculture Organization of the United Nations (http://faostat.fao.org). Eggplant is susceptible to many bacterial and fungal pathogens and insects, such as the *Verticillium dahlia* fungus and nematodes [3], which cause significant yield losses. As such, improving resistance to biotic and abiotic stresses is one of the main objectives of eggplant breeding programs. *S. torvum* Sw., commonly known as turkey berry, is a wild relative of eggplant and is resistant to root-knot nematodes and the most serious soil-borne diseases, such as those caused by *Ralstonia solanacearum*, *V. dahlia* Klebahn and *Fusarium oxysporum* f. sp. Melongenae [4]. Thus, turkey berry offers promising genetic resources for improving eggplant. Attempts have been made to introduce turkey berry resistance into eggplant through conventional breeding and biotechnological techniques; however, progress in this area has been limited [5,6,7,8,9].

In spite of their economic and experimental importance, genome-wide research resources are limited for eggplant and turkey berry, because of the lack of whole-genome sequences. The recent availability of comprehensive and high-quality *de novo* transcriptome assemblies of eggplant and turkey berry from short-read RNA-sequencing data [10] has allowed the identification of both housekeeping and regulatory gene families, such as the WRKY transcription factor (WRKY TF) gene family, one of the most important transcription factor families in plants [11]. WRKY proteins are involved in developmental processes and in responses to abiotic and biotic stresses [12]. Several WRKY proteins are involved in the regulation of plant growth and developmental processes, including trichome development [13], seed development and germination [14], embryogenesis [15,16] and leaf senescence [17]. WRKY proteins also play an important role in responses to various abiotic stresses [18], such as high salinity, drought and cold in Arabidopsis [19,20,21] and phytohormone treatments in rice (*Oryza sativa*) [22], and are involved in plant defense against biotic stresses, such as bacterial, fungal and viral pathogens [23,24,25,26].

The WRKY genes are primarily restricted to plants with the exception of several examples in protozoa [27]. Most WRKY proteins in this family contain at least one WRKY domain of ~60 amino acid residues [11]. The *N* terminus of the WRKY domain is characterized by a highly-conserved WRKYGQK motif, whereas the *C*-terminal region of the domain contains a metal-chelating zinc finger motif, either C–X_4-5_–C–X_22-23_–H–X–H (C_2_H_2_) or C–X_5–8_–C–X_25–28_–H–X_1-2_–C (C_2_HXC) [11]. The WRKYGQK amino acid sequences are involved in binding to the W-box (TTGACY) sequence, an element found in the promoters of many stress-related genes [28]. However, recent studies revealed that other factors are involved in WRKY binding to DNA in response to a specific stimulus [29,30]. WRKY proteins can be categorized into three groups according to their number of WRKY domains and the pattern of the zinc finger motif [11]. Group I WRKY TFs contain two WRKY domains (*N*-terminal and *C*-terminal) with distinct functions and include a C2H2 motif, whereas Group II and III WRKY TFs contain one WRKY domain with a C2H2 zinc finger motif and C2HXC zinc finger motif, respectively [11]. Based on the amino acid motifs outside the WRKY domain, the members in Group II can be divided into five subgroups: IIa–e [11].

In this study, we performed a genome-wide identification of WRKY genes in eggplant (SmelWRKYs) and turkey berry (StorWRKYs) using transcriptome data. Phylogenetic trees were constructed to classify WRKY genes and to evaluate their evolutionary relationships in a wide range of plant species. Detailed analyses of SmelWRKYs and StorWRKYs were performed, including identification of variants of the highly-conserved WRKY domain, analyses of evolutionary selective pressure and inference with the biological function**s** of these TFs based on Arabidopsis orthologs. Our results may provide candidate SmelWRKYs and StorWRKYs for genetic improvement of stress tolerance in eggplant and, probably, other Solanaceae plants.

## 2. Results and Discussion

### 2.1. Identification of the Eggplant and Turkey Berry WRKY Gene Families

As the first step in identifying important WRKY regulators that function in response to abiotic and biotic stresses [12], we sought to identify all SmelWRKY and StorWRKY family members in the recently released *de novo* transcriptome assemblies of eggplant and turkey berry [10]. To identify SmelWRKYs and StorWRKYs, we downloaded all transcript sequences from the National Center for Biotechnology Information (NCBI) [10]. With the assumption that the number of clusters assembled by Trinity reflects the total number of functional genes within the genome, the WRKY family members identified should approximate the entire family. Therefore, the longest transcript in each cluster was selected as the representative unigene. We then searched the assemblies with the longest predicted protein sequence for each unigene (File S1). A total of 50 and 62 non-redundant putative SmelWRKYs and StorWRKYs were identified by InterProScan [31]. All of these putative proteins were confirmed to contain a WRKY domain by the NCBI Conserved Domain Database (CDD), and 44 and 53 of these sequences were identified as complete SmelWRKYs and StorWRKYs, respectively (for Group I members, at least one complete WRKY domain was present; Table 1). The same analysis pipeline was used to identify WRKY TFs in *Amborella trichopoda*, greater duckweed (*Spirodela polyrhiza*), Arabidopsis, *Populus trichocarpa*, grape (*Vitis vinifera*), tomato, potato and hot pepper (*Capsicum annuum* L. Zunla-1), and the results are listed in Table 1. With our method, we identified 72 AtWRKYs (WRKY TFs in Arabidopsis), which are identical to those identified by PlnTFDB (3.0, http://plntfdb.bio.uni-potsdam.de/v3.0/fam_mem.php?family_id=WRKY&sp_id=ATH) and by PlantTFDB (v3.0, http://planttfdb.cbi.pku.edu.cn/family.php?sp=Ath&fam=WRKY). We also identified 81 SlWRKYs (WRKY TFs in tomato), which are identical to those identified by Huang *et al.* [32], confirming the robustness of our method.

Based on our results, the eggplant WRKY family may be the smallest reported Solanaceae WRKY family to date, but it is larger than the WRKY families of the basal angiosperm, *A. trichopoda*, and of greater duckweed (Table 1). In addition, the moss, *Physcomitrella patens* [33], the lycophyte, *Selaginella moellendorffii* [33], and castor bean (*Ricinus communis*) [34] have fewer WRKY TFs (37, 35 and 47, respectively). The size of the StorWRKY family is larger than that of the SmelWRKY family and is comparable to *Cucumis sativus* [35] and *Carica papaya* [25] with 55 and 66 WRKYs, respectively.

**Table 1:** The distribution of WRKY TFs from ten plant species. * For Group I, at least one of the two WRKY domains was identified as complete.

Species	Complete WRKY *	Partial WRKY	I	IIa	IIb	IIc	IId	IIe	III	Unassigned
*A. trichopoda*	29	3	6	2	4	4	3	4	4	2
*S. polyrhiza*	34	7	9	1	4	6	6	4	4	0
*A. thaliana*	72	0	14	3	8	17	7	9	13	1
*P. trichocarpa*	101	1	22	5	9	25	13	14	10	3
*V. vinifera*	57	2	12	3	8	15	6	6	6	1
*S. lycopersicum*	80	1	15	5	8	16	6	18	11	1
*S. tuberosum*	77	8	14	5	6	16	7	15	14	0
*C. annuum*	59	3	15	4	6	11	5	8	9	1
*S. melongena*	44	6	12	2	3	8	7	5	7	0
*S. torvum*	53	9	13	4	3	10	7	6	10	0

We noted that both the eggplant and turkey berry WRKY families were much smaller than those of their close *Solanum* relatives, tomato and potato (81 and 85 WRKY TFs, respectively; Table 1). The actual size of the SmelWRKY and StorWRKY families may be larger, as both were identified from transcriptome data. To address this possibility, we identified WRKY genes in hot pepper, for which the complete genome sequence is available [36]. Although the genome size of the hot pepper is about four-fold larger than that of its close relative, tomato [37], we identified only 62 WRKY TFs in pepper, which is much fewer than in tomato (Table 1). Moreover, it was recently reported that the ancestor of the Gentianales, Lamiales and Solanales likely contained ~65 WRKY TFs [38]. Therefore, the similar number of WRKY TFs in turkey berry and hot pepper suggests that StorWRKYs identified from transcriptome data likely represent essentially the entire WRKY gene pool in its genome and that >76% (50 out of 65) of SmelWRKYs were identified from the transcriptome data. These data (Table 1 and [38]) also suggest that, when compared with other asterid plants, tomato and potato may contain atypically large WRKY families.

### 2.2. Phylogenetic Analysis and Classification of the WRKY Gene Family

**Figure 1:**
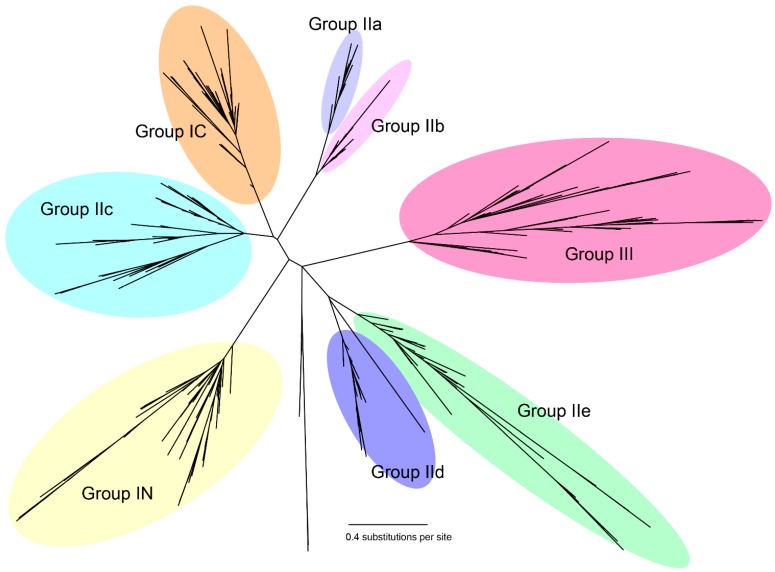
Phylogenetic tree of WRKY domains from ten species. The amino acid sequences of the WRKY domains were aligned, and the unrooted phylogenetic tree was constructed using the maximum likelihood method. The WRKY groups and subgroups are indicated. N, *N*-terminal domain; C, *C*-terminal domain.

To determine the phylogenetic relationships and groupings among WRKY TFs identified in this study, an unrooted phylogenetic tree was constructed based on the amino acid alignment of 712 complete WRKY domains (from 606 WRKY TFs) identified by NCBI CDD from *A. trichopoda*, Arabidopsis, hot pepper, *P. trichocarpa*, tomato, greater duckweed, potato, grape, eggplant and turkey berry (Figure 1). The two WRKY domains within the same WRKY protein were designated with N (Group IN) or C (Group IC) for the *N*- and *C*-terminal domain, respectively. Incomplete WRKY domain sequences were excluded, and the detailed alignments of the amino acid sequences are listed in File S2. A cladogram with tip labels and bootstrap values (>0.70) is presented in Figure S1. Based on the phylogenetic tree and the AtWRKY classifications, the WRKY genes were manually classified into groups and subgroups (Table 1). As reported in previous studies [27,32], the WRKY *N*- and *C*-terminal domains of Group I and the domains of Group III are monophyletic; however, those from Group II were not monophyletic and were grouped into three distinct clades, IIa + IIb, IIc and IId + IIe. Group IIc is closely related to Group IC and clustered with Group IIa + IIb, whereas Group IId + IIe clustered with Group III.

Inclusion of the WRKY sequences of the evolutionary basal angiosperm, *A. trichopoda*, and of a wide range of species not only reduced potential long-branch attractions during phylogenetic tree construction, but also provided valuable information about the evolutionary history and classification of WRKY TFs since the emergence of the basal angiosperm. Interestingly, all of the WRKY TFs were more similar to those in the same groups/subgroups in divergent species than they were to other WRKY proteins in the same species (Figure 1). As shown in Figure 1 and Table 1, together with results from previous studies [27,32,38,39], the interspersed distribution of the WRKY domains from all of these species in all of the groups and subgroups may suggest the existence of these groups/subgroups before the divergence of basal angiosperms and the expansion of particular groups and/or subgroups in some clades or species after the divergence from basal angiosperms (Figure S1).

In tomato, a distinct gene expansion event occurred in Group IIe [32], and we examined whether similar expansion events occurred in the groups and subgroups of eggplant and turkey berry. In eggplant, we identified 12 Group I, 25 Group II and seven Group III WRKY TFs, and the Group II proteins were subdivided into two Subgroup IIa, three Subgroup IIb, eight Subgroup IIc, seven Subgroup IId and five Subgroup IIe. In turkey berry, we identified 13 Group I, 30 Group II and 10 Group III WRKY TFs, and the Group II proteins were subdivided into four Subgroup IIa, three Subgroup IIb, 10 Subgroup IIc, seven Subgroup IId and six Subgroup IIe (Table 1). These patterns were similar to those of hot pepper, but obviously differed from those of tomato and potato, in which the Subgroup IIc, Group III and especially Subgroup IIe WRKY subfamilies are expanded (Figure S2), resulting in atypically large WRKY families.

Group I WRKY TFs contain two WRKY domains (*N*- and *C*-terminal) with distinct functions. However, in this study, several WRKY TFs with only one WRKY domain were phylogenetically grouped into Group IC or IN (Table 2). Moreover, several of the classified Group I proteins contained incomplete *C*-terminal WRKY domains, but none of them contained incomplete *N*-terminal WRKY domains. These results imply the gain and/or loss of WRKY domains during their evolution, which is consistent with the results of Zhang *et al.* [27], suggesting that Group I WRKY TFs, such as those found in algae, are the most evolutionarily ancient and that the members in Group II and Group III are the descendants derived from ancestral Group IC [27]. However, recent evidence suggests that the Group I WRKYs, and other WRKY TFs, originated from an ancestral Group IIc-like domain [29], and an analysis of *Lotus japonicus* and *Medicago truncatula* WRKY proteins indicates that some WRKYs in Group II originated from the *N*-terminal domain of Group I WRKYs [40]. These results indicate that the origins of the WRKY genes are complex.

**Table 2:** The distribution of subgroups of Group I WRKY TFs from ten plant species. IN, *N*-terminal WRKY domain; IC, *C*-terminal WRKY domain.

Species	Total Group I	With Two WRKY Domains			With One WRKY Domain	
		Complete Group I	Partial IC	Partial IN	IN	IC
*A. trichopoda*	6	–	–	–	–	–
*S. polyrhiza*	9	6	1	–	–	2
*A. thaliana*	14	12	1	–	–	1
*P. trichocarpa*	22	21	1	–	–	–
*V. vinifera*	12	11	–	–	–	1
*S. lycopersicum*	15	15	–	–	–	–
*S. tuberosum*	14	12	1	–	1	–
*C. annuum*	15	13	1	–	1	–
*S. melongena*	12	4	1	–	3	4
*S. torvum*	13	6	1	–	4	2

The number of Group I SmelWRKYs and StorWRKYs were comparable to their Solanaceae relatives; however, the number of SmelWRKYs and StorWRKYs that contained only one WRKY domain, but grouped with IC or IN, were considerably larger than in species with whole-genome sequences available (Table 2). Although the SmelWRKYs and StorWRKYs identified from transcriptome data likely represent the majority of WRKYs in these species (discussed above), our method may have introduced potential errors, such as RNA-sequencing artifacts, variations in splicing, incomplete splicing of introns and/or potential assembly errors that were due to highly polymorphic sites in some genes, all of which could have resulted in abnormally truncated WRKY proteins with only the *N*- or *C*-terminal WRKY domain detectable. This possibility is supported by the fact that most of the partial WRKY genes and genes for Group I WRKYs with only one WRKY domain would encode relatively short peptides and are not full-length unigenes (with both a start and stop codon) (Table S1 and Table S2).

### 2.3. Multiple Sequence Alignment of SmelWRKYs and StorWRKYs

**Figure 2:**
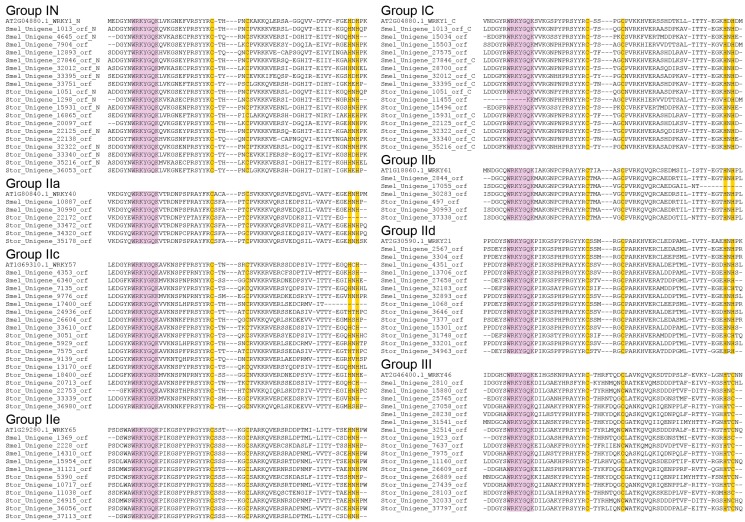
Multiple sequence alignment of the WRKY domains from eggplant and turkey berry WRKY genes. The suffixes “_N” and “_C” indicate the *N*- and *C*-terminal WRKY domain, respectively, of a specific WRKY gene from Group I. Alignment was performed using MEGA 6 [41]. Light purple indicates conserved WRKY amino acid domains, and orange indicates zinc-finger motifs.

The WRKY domains from eggplant and turkey berry WRKY TFs and several homologous Arabidopsis proteins were aligned in MEGA 6.0 [41] (Figure 2). For each group or subgroup, one Arabidopsis protein was selected, including *N*-terminal and *C*-terminal WRKY domains of AtWRKY1 (Group IN and IC, AT2G04880.1), AtWRKY40 (Group IIa, AT1G80840.1), AtWRKY61 (Group IIb, AT1G18860.1), AtWRKY57 (Group IIc, AT1G69310.1), AtWRKY21 (Group IId, AT2G30590.1), AtWRKY65 (Group IIe, AT1G29280.1) and AtWRKY46 (Group III, AT2G46400.1).

The WRKY domain contains the highly-conserved heptapeptide stretch, WRKYGQK, at its *N*-terminus followed by a zinc finger motif. Previous reports indicate that some plants contain variants of the WRKY domain, such as WRKYGKK, WRKYGEK and WRKYGSK [27]. In tomato, WRKYGKK is the most common variant, followed by WRKYGMK, WSKYGQK, WQKYGQK and WIKYGEN [32]. In turkey berry, however, WRKYGKK was the only variant, whereas in eggplant, WRKYGKK was the dominant variant and WRKYGEK was a minor variant (Figure 2). These variants were mainly identified in Group IIc, and Group IC and III also included one variant each. The prevalence of WRKYGKK over other variants, especially in Group IIc, was also observed in other species, such as Arabidopsis [11], tomato [32] and large soybean (*Glycine max*) [39]. Variation in the WRKYGQK motif can reduce, eliminate or alter DNA binding activity, and conversion of the conserved glutamine to lysine can reduce, but not eliminate, DNA binding [42]. In contrast, hot pepper WRKY1 (Capana08g000429), which carries the WRKYGKK motif and is a negative regulator of pathogen defense, can still recognize the W-box [43]. Therefore, variants of the consensus sequence may offer these SmelWRKYs and StorWRKYs the ability to recognize different *cis*-elements.

### 2.4. Evolutionary Selective Pressure in SmelWRKYs and StorWRKYs

**Figure 3:**
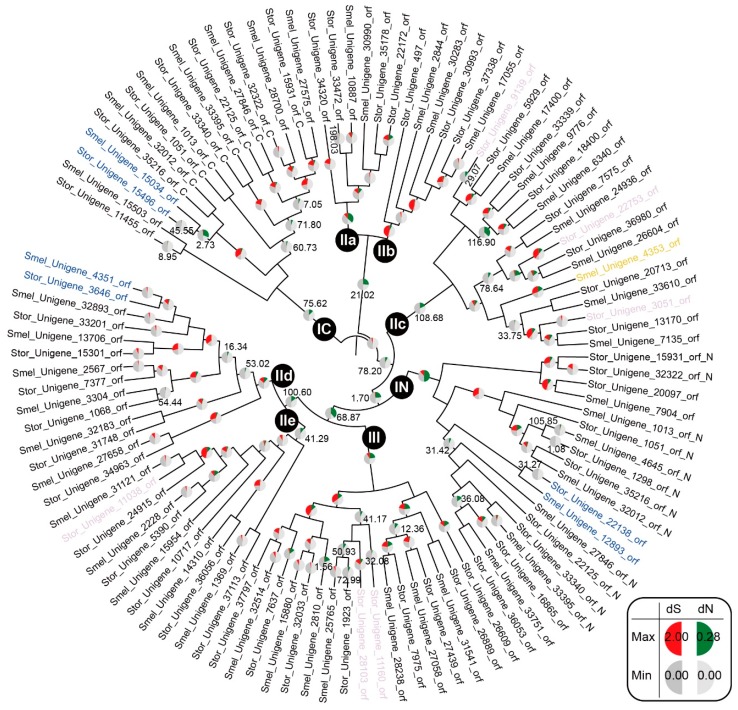
The branch-specific *d*N, *d*S and ω ratios of SmelWRKYs and StorWRKYs. The WRKY groups and subgroups are indicated. Branch-specific *d*S and *d*N are given if the *d*S value of that branch is between 0.1 and two or the ω ratio of that branch is >1. The left half of the circular inserts at each branch designates the branch-specific *d*S, and the size of the red sector is in proportion to *d*S, which ranged from zero to 2.00. The right half designates the branch-specific *d*N, and the size of the green sector is in proportion to *d*N, which ranged from zero to 0.28. The number on a branch is the ω ratio of that branch (only ω ratios >1 are given). Tips labeled with color are genes in some special orthologous groups identified by OrthoMCL (Table S2). Blue, eggplant- and turkey berry-specific orthologous groups; light purple, orthologous groups with genes from turkey berry, but without genes from eggplant; orange, orthologous groups with genes from eggplant, but without genes from turkey berry (Table 4).

Because they are involved in responses to abiotic and biotic stresses [12], the WRKY proteins may be subject to strong selective pressure imposed by these stresses. Previous studies have observed different selective pressures on different WRKY subgroups in large soybean [39], *L. japonicus* and *M. truncatula* [40]. Therefore, it is of great interest and importance to understand the molecular evolution of the WRKY gene family in eggplant and turkey berry. To detect whether selective pressure has affected WRKY subgroups in eggplant and turkey berry, an unrooted phylogenetic tree was constructed based on the amino acid alignment of complete SmelWRKY and StorWRKY domains (the alignments and tree files are included in File S3). As shown in Figure 3, the phylogenies of SmelWRKYs and StorWRKYs in the tree are in accordance with our WRKY classification results (Figure 2), further confirming the groupings.

The ω ratio (nonsynonymous substitution rate/synonymous substitution rate, *d*N/*d*S) is a measure of natural selection acting on a protein. Generally, values for ω of 1, >1 and <1 indicate neutral, positive and purifying selection, respectively. The branch-site model, which allows ω ratios to vary among sites and lineages simultaneously, appeared to be most suitable for describing evolutionary processes of the WRKY gene family [39]. In this model, the branches being tested for positive selection are referred to as the foreground branches, and all other branches on the tree are referred to as background branches. In this study, Groups IN, IC, IIa–e and III were selected as foreground branches, respectively, whereas the other groups were selected as the background branches. Likelihood ratio test (LRT) analysis revealed the presence of codons under positive selection in Groups IIc, IIe and III. When the Bayes empirical Bayes (BEB) method was implemented to calculate posterior probabilities for site classes, no positive selection sites were observed in Group IIe, and one positive selection site was found in Groups IIc and III at the 0.01 and 0.05 significance levels, respectively (Table 3). This result indicated that, in eggplant and turkey berry, Group IIc and Group III WRKY TFs may have been subjected to positive selection, whereas selective pressures in the other subgroups would seem to have been more conservative. Strong positive selective pressure was also observed in WRKY TFs from large soybean [39]. In that analysis, sites with high probabilities of having been under positive selection were found in Groups I, IIc, IIe and III, with sites in Group IIe and III appearing to have been under strong positive selective pressure. In contrast, Song *et al.* [40] found that Group III WRKY genes from *L. japonicus* and *M. truncatula* appear to be under purifying selection. Taken together, these results demonstrate that, in WRKY TFs, the natural selective pressures are likely to vary across different plant species and that subgroups have different evolutionary rates in particular species.

**Table 3:** Likelihood ratio tests for the branch-site models. ι, log likelihood values, 2Δι, the test statistic; ^a^ bold indicates *p* < 0.05 (based on the 50:50 mixture distribution of point mass 0 and ${\text{χ}}_{1}^{2}$); ^b^ amino acid sites estimated to have undergone positive selection by Bayes empirical Bayes analysis. * Posterior probability >95%, ** posterior probability >99%.

Group	ι (Foreground)	ι (Background)	2Δι	*p*-Value ^a^	Positive Selection Sites ^b^
IN	−4987.059421	−4987.867786	1.61673	2.50 × 10^−1^	8E *, 20N *, 34P *, 36F *
IC	−4990.205486	−4990.433633	0.45629	1.02 × 10^−1^	35A *
IIa	−4991.663923	−4991.663923	0.00000	5.00 × 10^−1^	none
IIb	−4991.663924	−4991.663923	−2.00 × 10^−6^	–	None
IIc	−4987.458167	−4989.190540	3.46475	**3.14 × 10^−2^**	6G **
IId	−4991.663923	−4991.663923	0.00000	5.00 × 10^−1^	none
IIe	−4988.849412	−4990.664638	3.63045	**2.84 × 10^−2^**	none
III	−4985.941961	−4988.138760	4.39360	**1.80 × 10^−2^**	5L *

To investigate how variable the evolutionary rates are in each group of SmelWRKYs and StorWRKYs, the branch-specific *d*N, *d*S and ω ratio were calculated using the free-ratios model (File S3), which assumes an independent ω ratio for each branch. As shown in Figure 3, most of the observed branch-specific ω ratios were <1, indicating purifying selective pressure in these WRKY domains and their ancestral branches. For the majority of branches leading to each group, atypically large ω ratios were observed, resulting from low *d*S values. Higher *d*N values in these branches relative to those in branches within each group also demonstrated that there is higher divergence at the amino acid level among groups than within each group. *d*S is recognized as an indicator of evolutionary rates. The high *d*S values (Figure 3) observed in each group indicate high evolutionary rates since the emergence of each group. However, for leaves of the tree (Figure 3), most *d*S values were <0.1 (Table S3), indicating that most WRKY domains from eggplant and turkey berry WRKY proteins have been conserved since their divergence from other WRKY genes.

### 2.5. Predicted Roles of SmelWRKY and StorWRKY Orthologs

To ascertain potential functions, we compared WRKY TFs by identifying orthologs and paralogs using OrthoMCL [44]. The protein database includes 606 WRKY TFs with complete WRKY domains (with at least one complete WRKY domain for Group I genes). In total, 73 orthologous groups were identified (File S4). We found that three of these orthologous groups (Cluster71–Cluster73) were eggplant- and turkey berry-specific clusters, and a high ω ratio (~2.73) was observed for the branch leading to Cluster72 (Smel_Unigene_15034_orf from eggplant and Stor_Unigene_15496_orf from turkey berry) (Figure 3), implying positive selection on this orthologous group in eggplant and turkey berry.

We further inspected the orthologous groups with genes from either eggplant or turkey berry. We identified six orthologous groups (Cluster22, Cluster26, Cluster28, Cluster32, Cluster39 and Cluster59) with genes from turkey berry, but without genes from eggplant (Table 4). These StorWRKYs may confer the stress-responsive ability to turkey berry that is absent in eggplant. However, the biological functions of the Arabidopsis orthologs of these StorWRKYs are not known, except for AtWRKY30 (ortholog of Stor_Unigene_11160_orf from Cluster39). Overexpression of AtWRKY30 is believed to enhance abiotic stress tolerance during the early growth stages in Arabidopsis [45]. Notably, we found only one orthologous group (Cluster21) with a gene from eggplant (Smel_Unigene_4353_orf), but without genes from turkey berry (Table 4). Interestingly, in Arabidopsis roots, the expression of AtWRKY23 (ortholog of Smel_Unigene_4353_orf from Cluster21) is upregulated in syncytia induced by the cyst nematode (*Heterodera schachtii*) and in giant cells induced by the root-knot nematode (*Meloidogyne incognita*) [46]. Eggplant is susceptible to root-knot nematodes, whereas turkey berry is tolerant to them [47]; and with respect to *H. schachtii*, tomato is susceptible and eggplant is less susceptible [48]. The presence of AtWRKY23 orthologs in eggplant and tomato may be important for nematode development. Moreover, a high *d*S value (~1.21, Figure 3 and Table S3) was observed in Smel_Unigene_4353_orf, indicating a high evolutionary rate since its divergence from other WRKYs.

Jasmonate and its derivatives are widely distributed in plants and affect a variety of processes, including responses to wounding and abiotic stress and defenses against insects and pathogens [49]. With publicly-available microarray datasets, Schluttenhofer *et al.* [38] identified six jasmonate-responsive Arabidopsis WRKY genes, AtWRKY7, AtWRKY20, AtWRKY26, AtWRKY45, AtWRKY48 and AtWRKY72, four of which (AtWRKY48, AtWRKY7, AtWRKY20 and AtWRKY72) were included in our clusters (Table 4, File S4). We found that AtWRKY7, which is also a calmodulin-binding TF [50], has two orthologs in eggplant, turkey berry, tomato and potato (File S4), indicating potential gene duplication in *Solanum*. Interestingly, we did not find an ortholog of WRKY72 in eggplant, turkey berry or three other Solanaceae species, although this TF is proposed to play a partially conserved role in basal defense in tomato and Arabidopsis [51].

Plant parasitic nematode infections generally occur as a result of root dysfunction and contribute to yield reductions. The most widespread and economically damaging nematode species include the sting nematode (*Belonolaimus longicaudatus*) and root-knot nematodes (*Meloidogyne* spp.). As noted above, eggplant is susceptible to root-knot nematodes, whereas turkey berry is tolerant. Recently, Bagnaresi *et al.* [47] identified 390 genes that were differentially expressed in turkey berry upon *M. incognita* infection. To investigate whether these root-knot nematode-responsive genes include WRKY TFs, we performed a BLASTN [52] search of the turkey berry 3' transcript library from Bagnaresi *et al.* [47] against the eggplant and turkey berry transcript of Yang *et al.* [10]. Corresponding transcript relationships were extracted using a reciprocal best hit method [52]. One of the 390 root-knot nematode-responsive genes was identified as StorWRKY (tor5_rep_c3275 in Bagnaresi *et al.* [47] and Stor_Unigene_36980 in Yang *et al.* [10]). The eggplant ortholog of Stor_Unigene_36980_orf is Smel_Unigene_26604_orf. To explore potential variations between these two orthologs, multiple sequence alignment of the WRKY domains from Cluster15 was conducted. When Stor_Unigene_36980_orf and Smel_Unigene_26604_orf were compared, we identified six variations at the nucleotide level (Figure S3), resulting in only two variations at the amino acid level, at the boundary of the domain (Figure S4). The ortholog of Stor_Unigene_36980_orf in Arabidopsis is AtWRKY75 (Table 4), which is also an oxalic acid-responsive AtWRKY gene [53]. Oxalic acid is an important pathogenicity determinant of necrotrophic phytopathogenic fungi [53], such as *Sclerotina sclerotiorum*, which causes Sclerotinia blight in eggplant [54]. Chen *et al.* [53] found that overexpression of AtWRKY75 in Arabidopsis enhances resistance to oxalic acid and to *S. sclerotiorum*.

**Table 4:** Selected orthologous groups of AtWRKYs, SmelWRKYs and StorWRKYs. ^a^ Oxalic acid-responsive AtWRKY genes [53]; ^b^ jasmonate-responsive AtWRKY genes [38].

Cluster	*A. thaliana*	*S. melongena*	*S. torvum*
Cluster1	AtWRKY46 ^a^	Smel_Unigene_32514_orf	Stor_Unigene_37797_orf
	AtWRKY53, AtWRKY41	Smel_Unigene_15880_orf	Stor_Unigene_32033_orf
Cluster6	AtWRKY7 ^b^	Smel_Unigene_13706_orf	Stor_Unigene_15301_orf
		Smel_Unigene_32893_orf	Stor_Unigene_33201_orf
Cluster7	AtWRKY20 ^b^	Smel_Unigene_33395_orf	Stor_Unigene_33340_orf
Cluster11	AtWRKY72 ^b^	–	–
Cluster15	AtWRKY75 ^a^	Smel_Unigene_26604_orf	Stor_Unigene_36980_orf
Cluster18	AtWRKY8, AtWRKY28 ^a^	Smel_Unigene_7135_orf	Stor_Unigene_13170_orf
Cluster19	AtWRKY6 ^a^	Smel_Unigene_30283_orf	Stor_Unigene_30993_orf
Cluster20	AtWRKY48 ^b^	Smel_Unigene_24936_orf	Stor_Unigene_7575_orf
Cluster21	AtWRKY23	Smel_Unigene_4353_orf	–
Cluster22	AtWRKY13	–	Stor_Unigene_9139_orf
Cluster26	AtWRKY24, AtWRKY43, AtWRKY56	–	Stor_Unigene_22753_orf
Cluster28	–	–	Stor_Unigene_3051_orf
Cluster32	AtWRKY29	–	Stor_Unigene_11038_orf
Cluster35	–	Smel_Unigene_10887_orf	Stor_Unigene_33472_orf
Cluster39	AtWRKY30	–	Stor_Unigene_11160_orf
Cluster40	AtWRKY62, AtWRKY67, AtWRKY38, AtWRKY66, AtWRKY64, AtWRKY63 ^a^	–	–
Cluster59	–	–	Stor_Unigene_28103_orf
Cluster71	–	Smel_Unigene_12893_orf	Stor_Unigene_22138_orf
Cluster72	–	Smel_Unigene_15034_orf	Stor_Unigene_15496_orf
Cluster73	–	Smel_Unigene_4351_orf	Stor_Unigene_3646_orf

## 3. Experimental Section

### 3.1. Dataset Collection

The annotated genome sequences of *S. lycopersicum* and *S. tuberosum* were downloaded from Sol Genomics Network (ITAG 2.3 http://solgenomics.net/organism/solanum_lycopersicum/genome and PGSC DM 3.4; http://solgenomics.net/organism/Solanum_tuberosum/genome, respectively). The annotated genome sequences of Arabidopsis, greater duckweed, *A. trichopoda* and hot pepper were downloaded from the Arabidopsis Information Resource (TAIR Release 10, ftp://ftp.arabidopsis.org/home/tair/Genes/TAIR10_genome_release), Mockler Lab (http://spirodelagenome.org/), the Amborella Genome Database (http://amborella.huck.psu.edu/) and the Pepper Genome Database (http://peppersequence.genomics.cn/page/species/download.jsp), respectively. The annotated genome sequences of *V. vinifera* and *P. trichocarpa* were downloaded from JGI phytozome v9.0 (ftp://ftp.jgi-psf.org/pub/compgen/phytozome/v9.0/Vvinifera and ftp://ftp.jgi-psf.org/pub/compgen/phytozome/v9.0/Ptrichocarpa/). To obtain high-quality representative gene sets for the above genomes, the longest translation form was chosen to represent each gene, and gene models with open reading frames shorter than 150 bp in the genomes were removed. Eggplant and turkey berry transcripts were downloaded from the NCBI Transcriptome Shotgun Assembly database under Accession Numbers GBEF00000000 (eggplant) and GBEG00000000 (turkey berry) [10]. The longest transcript in each cluster was selected as the representative unigene that was subjected to annotation for open reading frames.

### 3.2. Structural Annotation of Eggplant and Turkey Berry Unigenes

A BLASTX [55] search with a cut-off E-value ≤ 1 × 10^−5^ was performed against public protein databases, including the NCBI non-redundant database, SwissProt [56] and the potato (PGSC DM 3.4) and tomato (ITAG 2.3) protein sets. The coding sequences (CDSs) of all putative unigenes were extracted from the BLASTX results (homologous approach) with a minimum 150-bp cutoff value and the priority order of SwissProt, *Solanum* (tomato and potato) protein datasets and the NCBI database if conflicting results were obtained. ESTSCAN software [57] was also used to determine the direction of sequences that did not align to any of the databases, and CDSs shorter than 150 bp were removed. To avoid missing potential coding transcripts, the unigenes for which CDSs were not predicted by either homologous or ESTSCAN approaches were subjected to an in-house script, which, like most gene prediction programs, uses fifth-order hidden Markov chains to model coding regions [58]. Again, the CDSs shorter than 150 bp were removed. The resultant CDSs extracted from the eggplant and turkey berry unigenes were translated into amino acid sequences with the standard codon table.

### 3.3. WRKY Gene Identification

InterProScan version 4.5 [31] was used to scan protein sequences against the protein signatures from InterPro to infer protein families and domains for the protein-coding genes. The integrated Pfam database was selected, and then, the default parameters were used. The genes with the WRKY DNA-binding domain (PF03106) were recognized as candidate WRKY TFs. Subsequently, a FASTA file of the candidate WRKY protein sequences was submitted to the NCBI CDD to confirm the presence of the WRKY domain and to identify complete and partial WRKY domains. This process was performed for each proteome used in our analysis, including *A. trichopoda*, Arabidopsis, hot pepper, *P. trichocarpa*, tomato, greater duckweed, potato, *V. vinifera*, eggplant and turkey berry.

### 3.4. WRKY Gene Classification

The protein sequences of the complete WRKY domains identified by the NCBI CDD were collected using an in-house Perl script. Multiple sequence alignment of these domain sequences from all ten plant species was performed using MUSCLE v3.8.31 [59] with default parameters, and the alignments were then subjected to maximum likelihood phylogenetic analyses using PhyML3.1 [60]. The parameters used in the tree construction were the JTT (Jones-Taylor-Thornton) model plus gamma-distributed rates, and bootstrap values were calculated using the aLRT (average Likelihood Ratio Test) model. The trees were visualized and optimized in Figtree (http://tree.bio.ed.ac.uk/software/figtree/). The WRKY genes were classified into different groups and subgroups based on the Arabidopsis WRKY classifications.

### 3.5. Selection Pressure Analyses

The amino acid sequences of complete WRKY domains from eggplant and turkey berry WRKY TFs were used to estimate a phylogenetic tree as described above. The amino acid sequence alignments were then converted into the corresponding CDS alignments, which were, together with the tree, used to estimate *d*S and *d*N and to detect positive selection using CODEML in the PAML 4.8 package in a maximum likelihood framework. The branch-specific *d*N, *d*S and ω ratios were calculated using the free-ratios model and an F3×4 codon frequency model.

The recommended branch-site test of positive selection was applied to detect positive selection affecting a few particular sites along selected lineages. We compared the null hypothesis, in which sites may evolve either neutrally (ω = 1) or under purifying selection (ω < 1), with the alternative hypothesis, which allows sites to be under positive selection (ω > 1). We then conducted the LRT analysis. The null distribution was a 50:50 mixture of chi-squared distributions with 1 degree of freedom and a point mass at zero; therefore, the *p*-values calculated based on this mixture distribution were used to guide against violations of model assumptions. Posterior probabilities were calculated using the BEB method. The nodes were considered to have undergone positive selection if they showed a statistically-significant LRT, and positively selected sites were identified in the BEB analysis.

### 3.6. Identification of Gene Orthologous Groups

The translated eggplant and turkey berry WRKY TF amino acid sequences were pooled into a WRKY TF protein database with sequences from another eight plant species: *A. trichopoda*, greater duckweed, Arabidopsis, *P. trichocarpa*, *V. vinifera*, tomato, potato and hot pepper [36]. Only sequences with at least one complete WRKY domain were retained (the sequences that generated the WRKY domains in Figure 1). Self-to-self BLASTP [55] was conducted for all amino acid sequences with a cut-off *E*-value of 1 × 10^−5^, and hits with identity <30% and coverage <30% were removed. Orthologous groups were constructed from the BLASTP results with OrthoMCL v2.0.9 [44] using default settings.

## 4. Conclusions

In this study, we identified 50 SmelWRKYs and 62 StorWRKYs, and all of them could be classified into three groups (I–III). The SmelWRKY and StorWRKY families contain ~76% and ~95% of the number of WRKYs found in other sequenced asterid species, respectively. Different selection constraints could have affected the evolution of these groups. Sites with high probabilities of having been under positive selection were found in a subgroup of Group II (Group IIc) and Group III. Most WRKY domains from eggplant and turkey berry WRKY proteins are conserved and have evolved at low rates since their divergence. We also identified several pathogen resistance-related SmelWRKYs and StorWRKYs, providing possible candidate genetic resources for improving stress tolerance in eggplant and, probably, other Solanaceae plants. Overall, our results not only further our understanding of the evolutionary processes of eggplant and turkey berry WRKY genes, but also facilitate future functional genomics studies in these economically and genetically important crops.

## Supplementary Materials

Supplementary materials can be found at http://www.mdpi.com/1422-0067/16/04/7608/s1.
