# Supplementary material for: The WRKY Transcription Factor Genes in Eggplant (Solanum melongena L.) and Turkey Berry (Solanum torvum Sw.)
Source: Int J Mol Sci. 2015 Apr 7;16(4):7608–26. doi: 10.3390/ijms16047608 (PMC4425038; doi:10.3390/ijms16047608)
Supplement: Supplementary file 1 [file ijms-16-07608-s001.zip › ijms-69776-Supplementary Information/ijms-69776-supplementary.pdf]

Supplementary Information

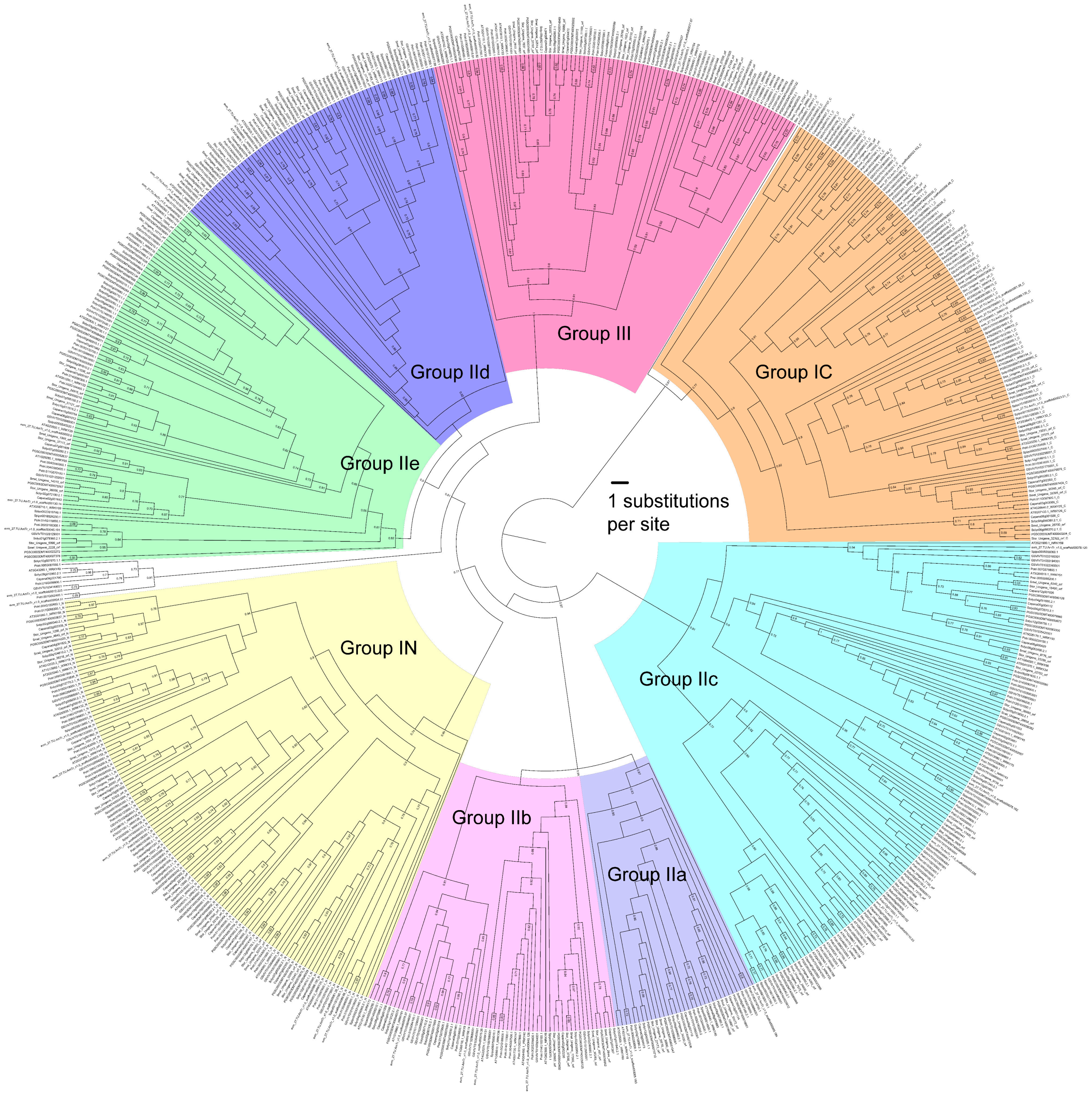

Figure S1. Phylogenetic tree of WRKY domains from ten species. The amino acid sequences of the WRKY domains were aligned, and the phylogenetic tree was constructed using the maximum likelihood method. The numbers beside the branches represent bootstrap values (only the values larger than 0.70 were shown). Each WRKY groups and subgroups are labeled accordingly. Group I proteins with the suffix "N" and "C" indicate the *N*- and *C*-terminal WRKY domains, respectively.

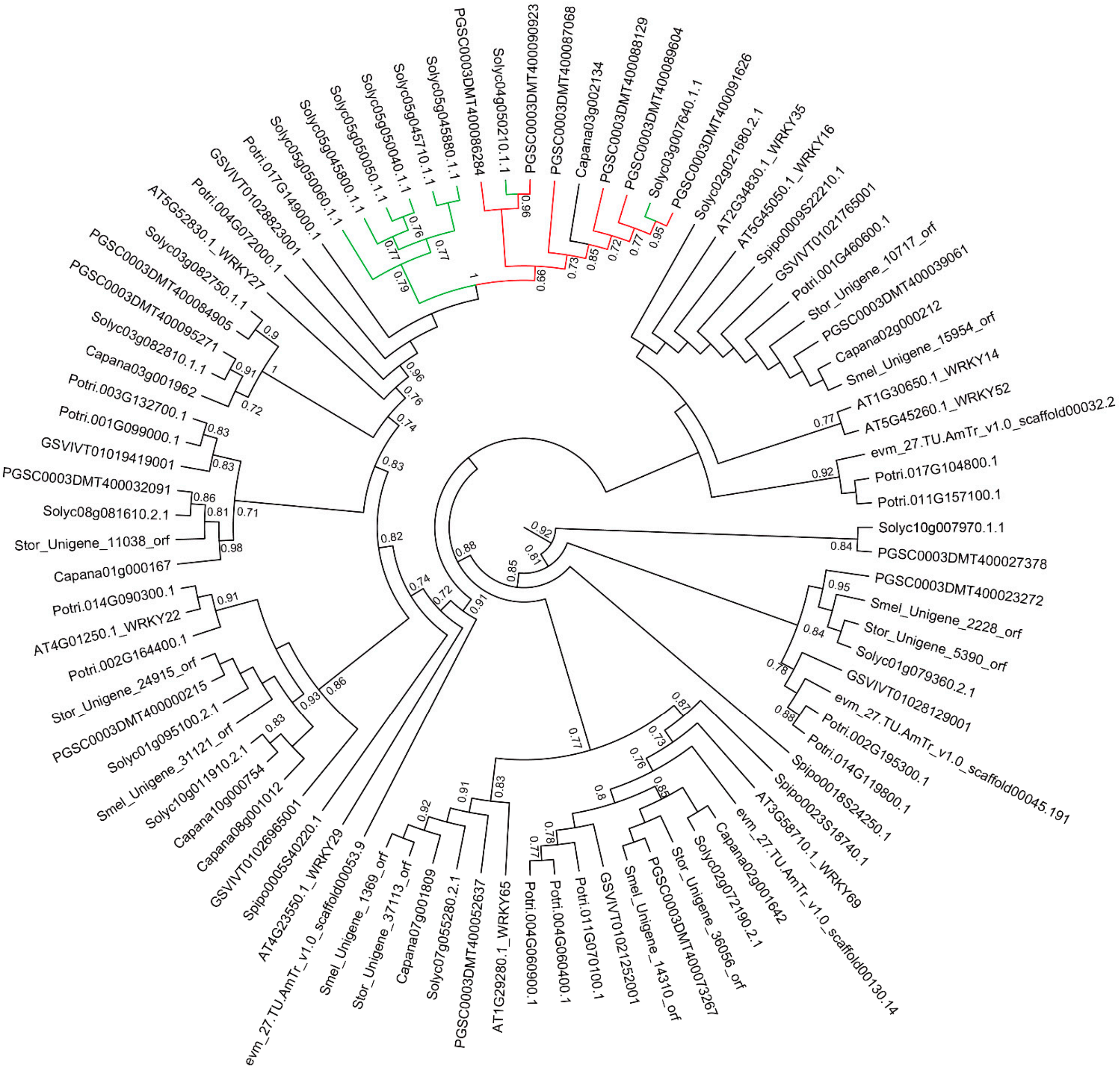

Figure S2. Phylogenetic tree of WRKY domains of group III WRKY TFs from ten species. This tree was extracted from Figure S1, and the numbers beside the branches represent bootstrap values. The distinct gene expansion in tomato and potato is indicated by coloring the subclade with green and red color respectively.

```
AT5G13080.1               LDDGYRWRKYGQKAVKNNKFPRSYYRCTYGGCNVKKQVQRLTVDQEVVVTTYEGVHSHPIEKSTENFEHIL
Potri.001G058800.1        LDDGYRWRKYGQKAVKNNKFPRSYYRCTHQGCNVKKQVQRLTKDEGIVVTTYEGTHSHQIEKSTDNFEHIL
Potri.T043800.1           LDDGYRWRKYGQKAVKNNKFPRSYYRCTYQGCTVKKQVQRLTKDEGVVVTTYEGMHSHPIEKSNDNFEHIL
Potri.003G169100.1        LDDGYRWRKYGQKAVKNNKFPRSYYRCTHQGCNVKKQVQRLTKDEGVVVTTYEGMHSHQIEKSPDNFEHIL
Potri.001G328000.1        LDDGYRWRKYGQKAVKNNKFPRSYYRCTYQGCNVKKQVQRLTKDEGVVVTTYEGMHTHPIEKPNDNFEHIL
GSVIVT01033063001         LDDGYRWRKYGQKAVKNNKFPRSYYRCTHQGCNVKKQVQRLSKDESIVVTTYEGVHTHPIEKPTDNFEHIL
GSVIVT01010525001         LDDGYRWRKYGQKAVKNNRFPRSYYRCTHQGCNVKKQVQRLSKDEGIVVTTYEGMHSHQIEKSTDNFEHIL
Capana00g001033           LDDGYRWRKYGQKAVKNNKFPRSYYRCTHQGCNVKKQVQRLSKDEGVVVTTYEGMHSHPIDKSTDNFEQIL
PGSC0003DMT400056352      LDDGYRWRKYGQKAVKNNKFPRSYYRCTHQGCNVKKQVQRLSKDEEVVVTTYEGMHSHPIDKSTDNFEHIL
Solyc05g015850.2.1        LDDGYRWRKYGQKAVKNNKFPRSYYRCTHQGCNVKKQVQRLSKDEEVVVTTYEGMHSHPIDKSTDNFEHIL
Stor_Unigene_36980_orf    LDDGYRWRKYGQKAVKNNKFPRSYYRCTHQGCNVKKQVQRLSKDEEVVVTTYEGMHSHPIDKSTDNFEHIL
Smel_Unigene_26604_orf    LDDGYRWRKYGQKAVKNNKFPRSYYRCTHQGCNVKKQVQRLSKDEEVVVTTYEGMHSHPIDKSTDNFDHMS
```

Figure S3. Multiple sequence alignments of amino acid of WRKY domains from orthologous group Cluster15. Amino acids labeled with red on Smel_Unigene_26604_orf indicate variations to Stor_Unigene_36980_orf. Light purple indicates conserved WRKY amino acid domains, and orange indicates zinc-finger motifs.

```
AT5G13080.1                CTTGATGATGGTTATCGATGGAGGAAATATGGCCAAAAGGCCGTCAAGAA
Potri.T043800.1            CTTGATGATGGTTATCGGTGGAGGAAGTATGGACAGAAGGCAGTGAAGAA
Potri.001G328000.1         CTTGATGATGGTTATAGATGGAGGAAGTATGGACAAAAGGCAGTGAAGAA
Potri.001G058800.1         CTTGATGATGGCTATAGATGGAGAAAGTATGGTCAAAAGGCAGTGAAGAA
Potri.003G169100.1         CTTGATGATGGATATCGATGGAGAAAGTATGGTCAAAAGGCCGTCAAGAA
GSVIVT01033063001          CTTGATGATGGATATAGATGGAGGAAGTACGGTCAAAAGGCAGTTAAGAA
GSVIVT01010525001          CTTGATGACGGTTACAGATGGAGGAAGTATGGGCAAAAAGCTGTCAAAAA
Capana00g001033            CTTGATGACGGTTATAGATGGAGGAAATATGGACAGAAGGCTGTCAAGAA
PGSC0003DMT400056352       CTTGATGATGGTTATAGATGGAGGAAATATGGACAAAAGGCTGTCAAAAA
Solyc05g015850.2.1         CTTGATGATGGTTATAGATGGAGGAAATATGGACAAAAAGCTGTCAAAAA
Stor_Unigene_36980_orf     CTTGATGATGGTTATAGATGGAGGAAATATGGACAGAAGGCTGTCAAAAA
Smel_Unigene_26604_orf     CTTGATGATGGTTATAGATGGAGGAAATATGGACAGAAGGCTGTCAAAAA
                           ******** ** **  * ***** ** ** ** ** ** ** ** ** **

AT5G13080.1                CAACAAGTTCCCTAGGAGTTACTATAGGTGTACATATGGAGGATGCAATG
Potri.T043800.1            CAACAAATTTCCGAGGAGCTATTACCGATGCACTTATCAAGGGTGCACTG
Potri.001G328000.1         CAACAAATTTCCTAGGAGCTATTACCGATGCACGTATCAAGGGTGTAACG
Potri.001G058800.1         CAACAAATTCCCCAGAAGCTACTATCGGTGCACACATCAAGGGTGTAATG
Potri.003G169100.1         CAACAAATTTCCCAGAAGCTACTATCGGTGTACACATCAAGGGTGCAACG
GSVIVT01033063001          CAACAAATTTCCAAGAAGCTACTACAGATGTACCCATCAGGGATGCAATG
GSVIVT01010525001          CAACAGGTTCCCAAGAAGCTATTATCGGTGTACACACCAAGGGTGCAATG
Capana00g001033            CAACAAATTCCCAAGGAGCTACTACCGATGCACGCATCAAGGATGTAACG
PGSC0003DMT400056352       CAACAAATTCCCAAGGAGCTATTATCGATGCACACATCAAGGATGTAACG
Solyc05g015850.2.1         CAACAAATTCCCAAGGAGCTATTATCGATGCACACATCAAGGATGTAACG
Stor_Unigene_36980_orf     CAACAAATTCCCAAGGAGCTACTACCGGTGCACACATCAAGGATGTAACG
Smel_Unigene_26604_orf     CAACAAATTCCCAAGGAGCTACTACCGGTGCACACATCAAGGATGTAACG
                           *****  ** ** ** ** ** **  * ** **  *    ** ** *  *

AT5G13080.1                TGAAGAAGCAAGTGCAAAGATTAACAGTGGACCAAGAAGTGGTCGTGACA
Potri.T043800.1            TAAAAAAGCAAGTCCAACGCTTAACCAAAGATGAGGGCGTCGTCGTGACC
Potri.001G328000.1         TAAAGAAGCAAGTCCAACGCCTAACCAAAGACGAAGGTGTTGTAGTGACG
Potri.001G058800.1         TGAAAAAGCAAGTTCAAAGATTAACTAAAGATGAAGGAATCGTGGTGACA
Potri.003G169100.1         TGAAGAAGCAGGTTCAGAGATTAACAAAAGACGAAGGAGTCGTGGTGACA
GSVIVT01033063001          TAAAGAAGCAGGTGCAACGCCTATCCAAAGATGAAAGCATTGTGGTGACT
GSVIVT01010525001          TGAAGAAGCAGGTTCAACGCCTATCAAAAGACGAAGGAATCGTGGTCACA
Capana00g001033            TGAAGAAACAAGTACAAAGGTTGTCCAAGGATGAAGGAGTAGTGGTAACT
PGSC0003DMT400056352       TGAAGAAACAAGTACAAAGATTATCCAAGGATGAAGAAGTAGTAGTAACT
Solyc05g015850.2.1         TGAAGAAACAAGTACAAAGATTATCAAAGGATGAAGAAGTAGTAGTTACT
Stor_Unigene_36980_orf     TGAAGAAACAAGTACAAAGGTTATCCAAGGATGAAGAGGTAGTAGTTACC
Smel_Unigene_26604_orf     TGAAGAAACAAGTACAAAGGTTATCGAAGGATGAAGAAGTAGTAGTAACC
                           * ** ** ** ** **  *  *  *    **  *     * ** ** **
```

Figure S4. *Cont.*

```
AT5G13080.1            ACCTACGAAGGAGTGCATTCGCATCCCATCGAGAAATCCACCGAAAACTT
Potri.T043800.1        ACTTACGAAGGGATGCATAGCCATCCTATAGAGAAGTCAAATGATAATTT
Potri.001G328000.1     ACTTACGAAGGAATGCACACCCATCCTATAGAGAAGCCAAATGATAATTT
Potri.001G058800.1     ACCTATGAAGGAACGCATTCTCATCAGATTGAAAAGTCTACAGATAACTT
Potri.003G169100.1     ACCTATGAAGGAATGCATTCTCATCAGATCGAAAAGTCTCCAGATAACTT
GSVIVT01033063001      ACTTATGAAGGGGTGCACACACATCCAATCGAGAAGCCCACCGACAATTT
GSVIVT01010525001      ACTTATGAAGGCATGCATTCGCATCAGATCGAGAAATCTACTGATAACTT
Capana00g001033        ACTTATGAAGGCATGCATTCACATCCCATTGACAAGTCTACCGATAACTT
PGSC0003DMT400056352   ACTTATGAAGGCATGCATTCACATCCCATTGACAAATCTACCGATAACTT
Solyc05g015850.2.1     ACTTATGAAGGCATGCATTCACATCCAATTGACAAATCTACGGATAACTT
Stor_Unigene_36980_orf ACTTATGAAGGCATGCATTCACATCCCATTGACAAGTCTACCGATAACTT
Smel_Unigene_26604_orf ACTTATGAAGGCATGCATTCACATCCCATTGACAAGTCTACCGATAACTT
                       ** ** *****   ***    ****  ** ** **  *    ** ** **

AT5G13080.1            CGAGCATATTCTC
Potri.T043800.1        TGAACATATATTA
Potri.001G328000.1     TGAACATATCTTG
Potri.001G058800.1     TGAGCATATCTTG
Potri.003G169100.1     CGAGCATATCTTG
GSVIVT01033063001      CGAACATATCTTG
GSVIVT01010525001      TGAGCATATCTTG
Capana00g001033        TGAGCAGATTTTG
PGSC0003DMT400056352   TGAGCACATTTTG
Solyc05g015850.2.1     TGAGCACATTTTG
Stor_Unigene_36980_orf TGAGCACATTTTG
Smel_Unigene_26604_orf TGACCACATGTCG
                        ** ** **
```

Figure S4. Multiple sequence alignments of coding sequences of WRKY domains from orthologous group Cluster15. Nucleotides labeled with red on Smel_Unigene_26604_orf indicate variations to Stor_Unigene_36980_orf. Light purple indicates conserved WRKY amino acid domains, and orange indicates zinc-finger motifs. * = this column of the alignment contains identical bases in all sequences.

Table S1. A list of eggplant WRKY domain containing unigenes.

Unigene ID	Unigene Length	CDS Info. *	CDS Integrity **	Protein Length	Class ***	WRKY Domain Info. ****
Smel_Unigene_1013	1522	[345,1517,-]	01	391	I	[100,158,59,-]<br>[276,334,59,-]
Smel_Unigene_27846	2013	[1,1785,-]	10	595	I	[323,381,59,-]<br>[536,594,59,-]
Smel_Unigene_32012	1503	[304,1500,-]	01	399	I	[107,165,59,-]<br>[286,344,59,-]
Smel_Unigene_33395	2235	[124,1872,+]	01	583	I	[222,280,59,-]<br>[398,456,59,-]
Smel_Unigene_4645	1864	[774,1760,-]	01	329	IN	[140,194,55,-]<br>[299,320,22,C]
Smel_Unigene_12893	455	[87,413,-]	00	109	IN	[1,60,60,-]
Smel_Unigene_33751	2268	[982,1554,-]	00	191	IN	[1,58,58,-]
Smel_Unigene_7904	1133	[146,988,-]	10	281	IN	[193,249,57,-]
Smel_Unigene_15034	490	[8,400,+]	01	131	IC	[49,106,58,-]
Smel_Unigene_15503	501	[29,403,-]	00	125	IC	[43,102,60,-]
Smel_Unigene_27575	2118	[1160,1789,+]	11	210	IC	[42,100,59,-]
Smel_Unigene_28700	2270	[1341,1877,+]	01	179	IC	[20,78,59,-]
Smel_Unigene_10887	454	[88,453,+]	00	122	IIa	[17,76,60,-]
Smel_Unigene_30990	1324	[71,991,+]	01	307	IIa	[133,191,59,-]
Smel_Unigene_17055	377	[76,375,+]	00	100	IIb	[50,100,51,-]
Smel_Unigene_2844	1943	[221,1771,-]	10	517	IIb	[284,342,59,-]
Smel_Unigene_30283	1952	[61,1695,+]	11	545	IIb	[298,358,61,-]
Smel_Unigene_17400	288	[1,288,-]	00	96	IIc	[47,96,50,-]
Smel_Unigene_24936	358	[39,320,+]	00	94	IIc	[30,89,60,-]
Smel_Unigene_26604	213	[1,210,+]	00	70	IIc	[1,59,59,-]
Smel_Unigene_33610	1312	[322,1137,-]	01	272	IIc	[123,180,58,-]
Smel_Unigene_4353	1025	[394,678,-]	00	95	IIc	[32,89,58,-]
Smel_Unigene_6340	681	[156,371,-]	00	72	IIc	[13,71,59,-]
Smel_Unigene_7135	502	[78,482,-]	00	135	IIc	[42,101,60,-]
Smel_Unigene_9776	671	[167,424,-]	01	86	IIc	[13,72,60,-]
Smel_Unigene_13706	1640	[377,1480,-]	11	368	IId	[293,352,60,-]
Smel_Unigene_2567	1003	[274,825,-]	11	184	IId	[115,175,61,-]
Smel_Unigene_27658	1248	[22,969,+]	10	316	IId	[240,296,57,-]
Smel_Unigene_32183	517	[3,326,+]	01	108	IId	[31,89,59,-]
Smel_Unigene_32893	1257	[172,1146,-]	11	325	IId	[251,311,61,-]
Smel_Unigene_3304	1212	[3,935,+]	01	311	IId	[242,302,61,-]
Smel_Unigene_4351	664	[18,407,+]	01	130	IId	[56,116,61,-]
Smel_Unigene_1369	534	[147,506,-]	00	120	IIe	[43,99,57,-]
Smel_Unigene_14310	300	[10,300,-]	00	97	IIe	[34,94,61,-]
Smel_Unigene_15954	501	[240,500,-]	00	87	IIe	[18,78,61,-]
Smel_Unigene_2228	870	[27,386,+]	10	120	IIe	[59,118,60,-]
Smel_Unigene_31121	1431	[71,1129,+]	11	353	IIe	[173,233,61,-]

Table S1. *Cont.*

Unigene ID	Unigene Length	CDS Info. *	CDS Integrity **	Protein Length	Class ***	WRKY Domain Info. ****
Smel_Unigene_15880	1559	[288,1370,-]	11	361	III	[133,192,60,-]
Smel_Unigene_25765	1369	[35,616,+]	10	194	III	[135,194,60,-]
Smel_Unigene_27058	992	[414,899,-]	10	162	III	[100,160,61,-]
Smel_Unigene_2810	944	[350,616,-]	00	89	III	[14,76,63,-]
Smel_Unigene_28238	992	[29,514,+]	10	162	III	[102,162,61,-]
Smel_Unigene_31541	838	[6,599,+]	10	198	III	[111,173,63,-]
Smel_Unigene_32514	2322	[1306,2298,-]	01	331	III	[106,167,62,-]
Smel_Unigene_11729	296	[47,226,+]	00	60	p	[1,40,40,N]
Smel_Unigene_17684	247	[74,247,+]	00	58	p	[20,58,39,C]
Smel_Unigene_18151	364	[1,168,-]	00	56	p	[36,56,21,C]
Smel_Unigene_26098	236	[8,235,-]	01	76	p	[54,75,22,C]
Smel_Unigene_26527	221	[2,220,+]	00	73	p	[1,40,40,N]
Smel_Unigene_7539	273	[76,267,-]	11	64	p	[42,63,22,C]

* CDS information, format: [start, end, phase]; ** CDS completeness: 00, without both start and stop codon; 01, only stop codon; 10, only start codon; 11, without both start and stop codon; *** Classification information: I, Group I with completed *N*-terminal and *C*-terminal WRKY domains; IN, Group I with incomplete or without *C*-terminal WRKY domain; IC, Group I with incomplete or without *N*-terminal WRKY domain; p, partial; **** WRKY domain information identified by the NCBI CDD, format: [start, end, domain length, completeness]. For completeness, there are three categories: N, WRKY domain with incomplete *N*-terminal; C, WRKY domain with incomplete *C*-terminal; -, completed WRKY domain.

Table S2. A list of turkey berry WRKY domain containing unigenes.

Unigene ID	Unigene Length	CDS Info.*	CDS Integrity **	Protein Length	Class ***	WRKY Domain Info. ****
Stor_Unigene_1051	1660	[44,1291,+]	01	416	I	[125,183,59,-]<br>[301,359,59,-]
Stor_Unigene_15931	1981	[316,1782,-]	11	489	I	[157,215,59,-]<br>[322,380,59,-]
Stor_Unigene_22125	2755	[241,2481,+]	11	747	I	[323,381,59,-]<br>[536,594,59,-]
Stor_Unigene_32322	2086	[247,1719,+]	01	491	I	[161,219,59,-]<br>[332,390,59,-]
Stor_Unigene_33340	2458	[206,1954,+]	01	583	I	[222,280,59,-]<br>[398,456,59,-]
Stor_Unigene_35216	1994	[156,1688,+]	11	511	I	[223,281,59,-]<br>[397,455,59,-]
Stor_Unigene_1298	2558	[889,1770,+]	11	294	IN	[97,151,55,-]<br>[264,285,22,C]
Stor_Unigene_16865	768	[247,456,+]	00	70	IN	[10,68,59,-]
Stor_Unigene_20097	337	[102,323,+]	00	74	IN	[8,64,57,-]
Stor_Unigene_22138	270	[24,257,-]	00	78	IN	[7,66,60,-]
Stor_Unigene_36053	2279	[722,1333,+]	00	204	IN	[4,61,58,-]
Stor_Unigene_11455	896	[392,787,+]	11	132	IC	[3,51,49,-]
Stor_Unigene_15496	261	[2,247,-]	00	82	IC	[17,74,58,-]
Stor_Unigene_22172	283	[108,281,+]	10	58	IIa	[4,58,55,-]
Stor_Unigene_33472	1018	[199,894,-]	01	232	IIa	[102,162,61,-]
Stor_Unigene_34320	1718	[224,1294,+]	11	357	IIa	[167,227,61,-]
Stor_Unigene_35178	1480	[369,1421,-]	11	351	IIa	[166,226,61,-]
Stor_Unigene_30993	1853	[98,1750,-]	11	551	IIb	[304,364,61,-]
Stor_Unigene_37338	1446	[259,1293,+]	11	345	IIb	[110,170,61,-]
Stor_Unigene_497	1977	[200,1762,+]	10	521	IIb	[288,346,59,-]
Stor_Unigene_13170	508	[38,475,-]	00	146	IIc	[81,140,60,-]
Stor_Unigene_18400	678	[158,466,-]	00	103	IIc	[44,102,59,-]
Stor_Unigene_20713	1284	[297,1106,-]	01	270	IIc	[119,176,58,-]
Stor_Unigene_22753	217	[1,216,-]	00	72	IIc	[1,57,57,-]
Stor_Unigene_3051	997	[130,942,-]	01	271	IIc	[130,189,60,-]
Stor_Unigene_33339	556	[120,350,+]	01	77	IIc	[13,70,58,-]
Stor_Unigene_36980	683	[235,525,+]	11	97	IIc	[17,75,59,-]
Stor_Unigene_5929	653	[121,510,-]	01	130	IIc	[55,114,60,-]
Stor_Unigene_7575	475	[39,440,-]	00	134	IIc	[16,75,60,-]
Stor_Unigene_9139	706	[2,352,-]	10	117	IIc	[51,110,60,-]
Stor_Unigene_1068	1746	[485,1510,+]	11	342	IId	[273,333,61,-]
Stor_Unigene_15301	1570	[327,1412,-]	11	362	IId	[287,346,60,-]
Stor_Unigene_31748	1237	[30,980,+]	11	317	IId	[236,294,59,-]
Stor_Unigene_33201	1311	[244,1218,-]	11	325	IId	[251,311,61,-]
Stor_Unigene_34963	1279	[310,1242,-]	10	311	IId	[240,296,57,-]
Stor_Unigene_3646	752	[220,576,-]	01	119	IId	[45,105,61,-]

Table S2. *Cont.*

Unigene ID	Unigene length	CDS info.*	CDS integrity **	Protein length	Class ***	WRKY domain info. ****
Stor_Unigene_7377	800	[3,551,-]	10	183	IId	[115,175,61,-]
Stor_Unigene_10717	1450	[105,1289,-]	01	395	IIe	[170,230,61,-]
Stor_Unigene_11038	428	[98,394,-]	10	99	IIe	[25,83,59,-]
Stor_Unigene_24915	1538	[127,1185,+]	11	353	IIe	[173,233,61,-]
Stor_Unigene_36056	1248	[48,899,+]	00	284	IIe	[81,141,61,-]
Stor_Unigene_37113	941	[79,819,-]	01	247	IIe	[43,99,57,-]
Stor_Unigene_5390	669	[274,633,-]	10	120	IIe	[52,111,60,-]
Stor_Unigene_11160	467	[1,303,+]	00	101	III	[14,76,63,-]
Stor_Unigene_1923	1435	[304,1275,-]	11	324	III	[114,172,59,-]
Stor_Unigene_26609	241	[61,240,-]	00	60	III	[1,60,60,-]
Stor_Unigene_26889	990	[267,950,-]	10	228	III	[121,183,63,-]
Stor_Unigene_27439	1220	[91,579,+]	10	163	III	[101,161,61,-]
Stor_Unigene_28103	3031	[2094,2531,+]	10	146	III	[20,80,61,-]
Stor_Unigene_32033	1565	[199,780,+]	10	194	III	[133,194,62,-]
Stor_Unigene_37797	1214	[5,1015,+]	01	337	III	[112,173,62,-]
Stor_Unigene_7637	549	[40,294,+]	10	85	III	[25,85,61,-]
Stor_Unigene_7975	1438	[536,760,-]	00	75	III	[15,75,61,-]
Stor_Unigene_10178	300	[139,291,+]	00	51	p	[24,51,28,C]
Stor_Unigene_12458	477	[1,168,+]	01	56	p	[1,35,35,N]
Stor_Unigene_14442	454	[253,453,+]	00	67	p	[29,67,39,C]
Stor_Unigene_21457	528	[19,192,+]	10	58	p	[2,45,44,N]
Stor_Unigene_28410	276	[2,274,+]	00	91	p	[1,24,24,N]
Stor_Unigene_31491	214	[1,213,-]	00	71	p	[37,71,35,C]
Stor_Unigene_31743	1675	[1089,1481,+]	01	131	p	[1,37,37,N]
Stor_Unigene_33281	1445	[878,1321,-]	10	148	p	[127,148,22,C]
Stor_Unigene_4192	415	[30,401,+]	01	124	p	[1,40,40,N]

* CDS information, format: [start, end, phase]; ** CDS completeness: 00, without both start and stop codon; 01, only stop codon; 10, only start codon; 11, without both start and stop codon; *** Classification information: I, Group I with completed *N*-terminal and *C*-terminal WRKY domains; IN, Group I with incomplete or without *C*-terminal WRKY domain; IC, Group I with incomplete or without *N*-terminal WRKY domain; p, partial; **** WRKY domain information identified by the NCBI CDD, format: [start, end, domain length, completeness]. For completeness, there are three categories: N, WRKY domain with incomplete *N*-terminal; C, WRKY domain with incomplete *C*-terminal; -, completed WRKY domain.

Table S3. Estimation of *d*N, *d*S and ω ratio of SmelWRKYs and StorWRKYs.

Leaf	dN	dS	dN/dS(w)	Node *	dN	dS	dN/dS(w)
Stor_Unigene_26889_orf	0.0000	0.0000	0.0001	N1	0.0177	0.8898	0.0199
Smel_Unigene_31541_orf	0.0000	0.0000	0.1363	N2	0.0966	0.9118	0.1060
Stor_Unigene_27439_orf	0.0000	0.0000	0.0001	N3	0.0570	0.0046	12.3570
Smel_Unigene_27058_orf	0.0000	0.0923	0.0001	N4	0.0000	0.0433	0.0001
Stor_Unigene_7975_orf	0.0122	0.0774	0.1579	N5	0.0000	0.1113	0.0001
Smel_Unigene_28238_orf	0.0110	0.0641	0.1721	N6	0.0237	2.5381	0.0093
Stor_Unigene_26609_orf	0.0845	0.5543	0.1524	N7	0.0603	0.5436	0.1109
Stor_Unigene_34320_orf	0.0135	0.8318	0.0163	N8	0.2024	0.4427	0.4571
Smel_Unigene_30990_orf	0.0000	0.0000	0.0001	N9	0.0248	1.0316	0.0241
Stor_Unigene_35178_orf	0.0000	0.0000	0.0001	N10	0.0000	0.0931	0.0001
Stor_Unigene_22172_orf	0.0843	0.7752	0.1088	N11	0.0121	1.8066	0.0067
Stor_Unigene_33472_orf	0.0110	0.0001	198.0260	N12	0.0000	0.1275	0.0001
Smel_Unigene_10887_orf	0.0232	0.3602	0.0643	N13	0.0400	1.8384	0.0218
Stor_Unigene_497_orf	0.0000	0.0000	0.0001	N14	0.1425	0.0018	80.1208
Smel_Unigene_2844_orf	0.0000	0.0510	0.0001	N15	0.2122	10.5584	0.0201
Stor_Unigene_30993_orf	0.0000	0.0000	0.0001	N16	0.1715	0.0629	2.7269
Stor_Unigene_37338_orf	0.0000	0.0000	0.0001	N17	0.0617	2.2361	0.0276
Smel_Unigene_17055_orf	0.0000	0.1429	0.0001	N18	0.0293	1.4216	0.0206
Smel_Unigene_30283_orf	0.0000	0.0000	0.0001	N19	0.0864	3.7307	0.0232
Stor_Unigene_11455_orf	0.0115	0.0013	8.9456	N20	0.0177	0.8320	0.0213
Smel_Unigene_15503_orf	0.0000	0.0817	0.0001	N21	0.0309	0.7537	0.0409
Stor_Unigene_15496_orf	0.0116	0.0003	45.5465	N22	0.0292	0.6147	0.0475
Smel_Unigene_15034_orf	0.0000	0.0875	0.0001	N23	0.0298	0.8174	0.0364
Stor_Unigene_35216_orf_C	0.0000	0.0000	0.0001	N24	0.0115	1.1377	0.0101
Smel_Unigene_32012_orf_C	0.0000	0.0862	0.0001	N25	0.0256	0.0036	7.0479
Stor_Unigene_1051_orf_C	0.0000	0.0409	0.0001	N26	0.0280	0.0004	71.8012
Smel_Unigene_1013_orf_C	0.0000	0.0000	0.0001	N27	0.0203	0.0003	60.7312
Stor_Unigene_33340_orf_C	0.0000	0.0398	0.0001	N28	0.0000	0.0000	0.0001
Smel_Unigene_33395_orf_C	0.0000	0.0000	0.0001	N29	0.0701	0.0009	75.6164
Stor_Unigene_22125_orf_C	0.0000	0.0000	0.0001	N30	0.0000	0.1578	0.0001
Smel_Unigene_27846_orf_C	0.0000	0.0393	0.0001	N31	0.0232	0.6543	0.0354
Stor_Unigene_32322_orf_C	0.0000	0.1364	0.0001	N32	0.0771	0.2671	0.2886
Smel_Unigene_28700_orf	0.0000	0.0000	0.0001	N33	0.1167	0.1539	0.7583
Stor_Unigene_15931_orf_C	0.0000	0.0000	0.0001	N34	0.0441	0.0006	78.6418
Smel_Unigene_27575_orf	0.0000	0.0842	0.0001	N35	0.0266	2.9722	0.0090
Stor_Unigene_7575_orf	0.0000	0.0000	0.5603	N36	0.0498	0.6942	0.0717
Smel_Unigene_24936_orf	0.0000	0.0000	0.5963	N37	0.0508	1.5800	0.0322
Stor_Unigene_22753_orf	0.0611	1.5891	0.0385	N38	0.0135	0.0004	33.7535
Stor_Unigene_36980_orf	0.0000	0.0000	0.0001	N39	0.0000	0.0000	0.0001
Smel_Unigene_26604_orf	0.0000	0.0816	0.0001	N40	0.0483	0.3478	0.1390
Stor_Unigene_20713_orf	0.0000	0.0866	0.0001	N41	0.0487	1.2632	0.0385
Smel_Unigene_33610_orf	0.0000	0.0000	0.0001	N42	0.0976	4.6538	0.0210
Stor_Unigene_3051_orf	0.0000	0.1659	0.0001	N43	0.0439	0.9971	0.0440
Stor_Unigene_13170_orf	0.0000	0.0000	0.0001	N44	0.0511	0.9251	0.0552

Table S3. *Cont.*

Leaf	dN	dS	dN/dS(w)	Node *	dN	dS	dN/dS(w)
Smel_Unigene_7135_orf	0.0000	0.0433	0.0001	N45	0.2042	0.0018	116.8961
Smel_Unigene_4353_orf	0.0908	1.2117	0.0749	N46	0.0000	0.0000	0.0761
Stor_Unigene_9139_orf	0.0481	0.0017	29.0653	N47	0.0991	0.0009	108.6824
Stor_Unigene_5929_orf	0.0000	0.0000	0.0001	N48	0.0418	0.0005	78.1994
Smel_Unigene_17400_orf	0.0000	0.0000	0.0001	N49	0.0454	1.1667	0.0389
Stor_Unigene_33339_orf	0.0000	0.0000	0.0001	N50	0.0566	1.8073	0.0313
Smel_Unigene_9776_orf	0.0000	0.0412	0.0001	N51	0.0000	0.0000	0.0001
Stor_Unigene_18400_orf	0.0000	0.0000	0.0001	N52	0.0000	0.0000	0.0001
Smel_Unigene_6340_orf	0.0000	0.0000	0.0001	N53	0.0859	0.0024	36.0754
Stor_Unigene_15931_orf_N	0.0442	2.8690	0.0154	N54	0.0657	2.2230	0.0296
Stor_Unigene_32322_orf_N	0.0001	0.6394	0.0001	N55	0.0519	2.6081	0.0199
Stor_Unigene_20097_orf	0.0000	0.0000	0.0001	N56	0.0000	0.0532	0.0001
Smel_Unigene_7904_orf	0.0109	0.0420	0.2592	N57	0.0338	0.0011	31.4237
Smel_Unigene_27846_orf_N	0.0000	0.0000	0.0001	N58	0.1915	6.6708	0.0287
Stor_Unigene_22125_orf_N	0.0000	0.0740	0.0001	N59	0.0000	0.0000	0.0001
Smel_Unigene_33751_orf	0.0111	0.0140	0.7942	N60	0.0122	0.0899	0.1361
Stor_Unigene_36053_orf	0.0000	0.0687	0.0001	N61	0.0230	0.4343	0.0530
Stor_Unigene_16865_orf	0.0592	0.6269	0.0944	N62	0.0881	0.9627	0.0916
Stor_Unigene_33340_orf_N	0.0000	0.0084	0.0001	N63	0.0000	0.0804	0.0001
Smel_Unigene_33395_orf_N	0.0221	0.1326	0.1670	N64	0.0115	1.3594	0.0085
Stor_Unigene_22138_orf	0.0098	0.0003	31.2658	N65	0.0013	13.2657	0.0001
Smel_Unigene_12893_orf	0.0209	0.0461	0.4527	N66	0.2751	0.4718	0.5831
Smel_Unigene_1013_orf_N	0.0000	0.0000	0.0001	N67	0.1315	0.0772	1.7039
Stor_Unigene_1051_orf_N	0.0000	0.0000	0.0001	N68	0.0286	1.1958	0.0239
Smel_Unigene_4645_orf_N	0.0237	0.0002	105.8525	N69	0.0225	0.4784	0.0471
Stor_Unigene_1298_orf_N	0.0000	0.0000	1.0761	N70	0.0115	0.0002	54.4413
Stor_Unigene_35216_orf_N	0.0000	0.0243	0.0001	N71	0.0123	0.5441	0.0227
Smel_Unigene_32012_orf_N	0.0000	0.0170	0.0001	N72	0.0372	0.9986	0.0372
Stor_Unigene_34963_orf	0.0000	0.0000	0.0001	N73	0.0001	1.1639	0.0001
Smel_Unigene_27658_orf	0.0000	0.4023	0.0001	N74	0.0223	0.5030	0.0442
Stor_Unigene_31748_orf	0.0079	0.0374	0.2097	N75	0.0000	0.0000	0.0001
Smel_Unigene_32183_orf	0.0052	0.0949	0.0551	N76	0.0000	0.0000	0.0001
Stor_Unigene_1068_orf	0.0000	0.0919	0.0001	N77	0.0001	1.4173	0.0001
Smel_Unigene_3304_orf	0.0000	0.0000	0.0001	N78	0.0267	0.0016	16.3445
Stor_Unigene_7377_orf	0.0000	0.0352	0.0001	N79	0.0216	0.0004	53.0189
Smel_Unigene_2567_orf	0.0000	0.1021	0.0001	N80	0.0609	0.5548	0.1098
Stor_Unigene_15301_orf	0.0000	0.2631	0.0001	N81	0.0382	59.2233	0.0006
Smel_Unigene_13706_orf	0.0000	0.0000	0.0001	N82	0.0000	0.0000	0.0001
Stor_Unigene_33201_orf	0.0000	0.1487	0.0001	N83	0.0643	1.8555	0.0346
Smel_Unigene_32893_orf	0.0000	0.1706	0.0001	N84	0.0754	0.4074	0.1850
Stor_Unigene_3646_orf	0.0000	0.0178	0.0001	N85	0.0237	0.6605	0.0358
Smel_Unigene_4351_orf	0.0000	0.1308	0.0001	N86	0.0000	0.0000	0.0001
Stor_Unigene_37113_orf	0.0000	0.0000	0.0001	N87	0.0258	0.1606	0.1609

Table S3. *Cont.*

Leaf	dN	dS	dN/dS(w)	Node *	dN	dS	dN/dS(w)
Smel_Unigene_1369_orf	0.0000	0.1358	0.0001	N88	0.0108	1.0648	0.0101
Stor_Unigene_10717_orf	0.0000	0.0850	0.0001	N89	0.0000	0.3507	0.0001
Stor_Unigene_24915_orf	0.0000	0.0000	0.0001	N90	0.0566	0.0014	41.2887
Stor_Unigene_11038_orf	0.1314	2.7403	0.0479	N91	0.0814	0.0008	100.5951
Smel_Unigene_31121_orf	0.0000	0.1315	0.0001	N92	0.2153	0.0031	68.8743
Stor_Unigene_5390_orf	0.0000	0.0000	0.0001	N93	0.0112	0.4936	0.0227
Smel_Unigene_2228_orf	0.0000	0.1423	0.0001	N94	0.0000	0.0051	0.0001
Smel_Unigene_15954_orf	0.0000	0.0484	0.0001	N95	0.0112	0.6746	0.0166
Stor_Unigene_36056_orf	0.0000	0.0452	0.0001	N96	0.0000	0.0990	0.0001
Smel_Unigene_14310_orf	0.0000	0.0000	0.0001	N97	0.0745	1.6676	0.0446
Smel_Unigene_2810_orf	0.1061	0.0680	1.5607	N98	0.0470	0.0009	50.9324
Stor_Unigene_32033_orf	0.0000	0.1093	0.0001	N99	0.0359	6.1562	0.0058
Smel_Unigene_15880_orf	0.0000	0.2696	0.0001	N100	0.0375	0.0009	41.1727
Stor_Unigene_7637_orf	0.0830	0.1603	0.5180	N101	0.0794	1.3844	0.0573
Smel_Unigene_32514_orf	0.0000	0.2635	0.0001	N102	0.1244	0.3554	0.3500
Stor_Unigene_37797_orf	0.0000	0.0000	0.0001	N103	0.0000	0.0000	0.0001
Stor_Unigene_11160_orf	0.0184	0.0006	32.0792	N104	0.1254	0.8473	0.1480
Stor_Unigene_28103_orf	0.0457	0.6095	0.0749				
Stor_Unigene_1923_orf	0.0360	0.0005	72.9875				
Smel_Unigene_25765_orf	0.0000	0.1989	0.0001				

* Nodes information were recorded in File S3.
